# Supplementary material for: Accessing Structural, Electronic, Transport and Mesoscale Properties of Li-GICs via a Complete DFTB Model with Machine-Learned Repulsion Potential
Source: Materials (Basel). 2021 Nov 3;14(21):6633. doi: 10.3390/ma14216633 (PMC8585443; doi:10.3390/ma14216633)
Supplement: Supplementary file 1 [file materials-14-06633-s001.zip › materials-1424747-supplementary/SI/materials-1424747-supplementary.pdf]

Supporting Information - Accessing structural, electronic, transport and mesoscale properties of Li-GICs via a complete DFTB-model with machine-learned repulsion potential

Simon Anniés, Chiara Panosetti, Maria Voronenko, Dario Mauth, Christiane Rahe and Christoph Scheurer

September 2021

1 Initial states, final states, barriers in Table 3

Table 1: NEBS

Structure	**Initial State**	**Final State**	**Barrier**
LiC_{48} - stage 3	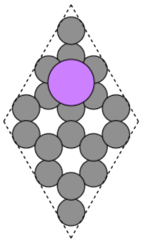	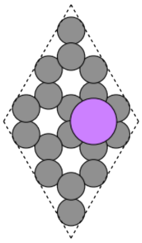	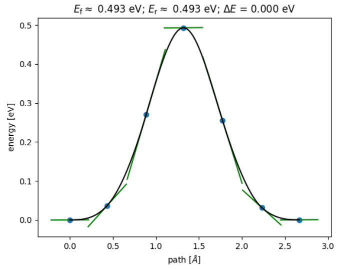
Li_2C_{48} - stage 3	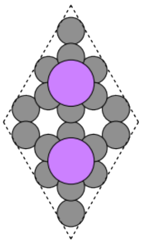	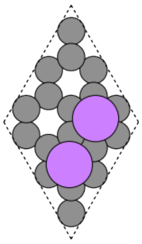	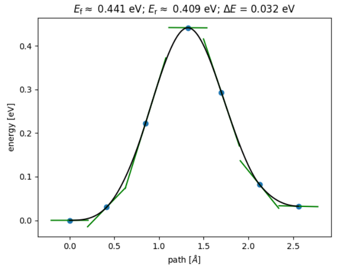
Li_3C_{48} - stage 3	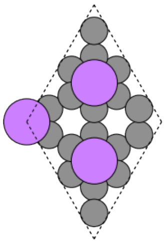	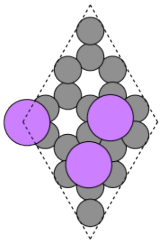	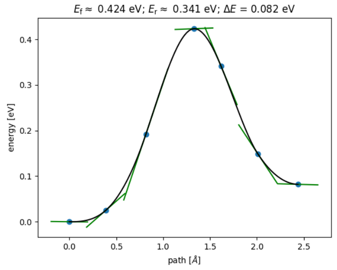

Table 2: NEBS - continuation

Structure	**Initial State**	**Final State**	**Barrier**
LiC_{36} - stage 2	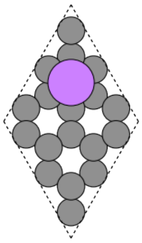	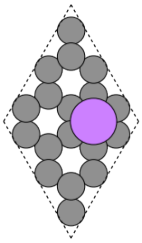	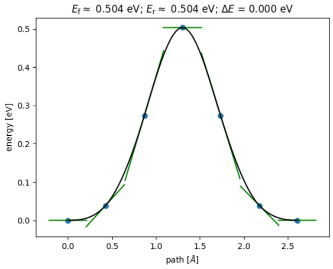
Li_2C_{36} - stage 2	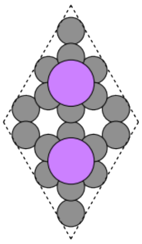	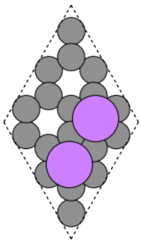	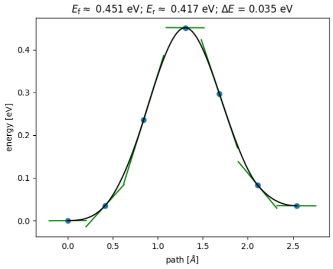
Li_3C_{36} - stage 2	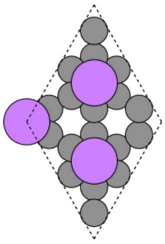	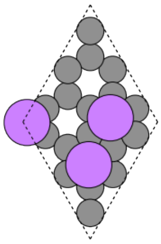	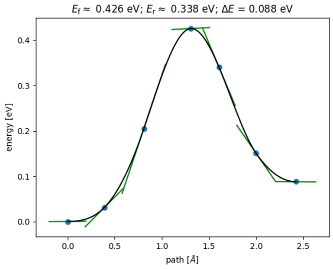
Li_2C_{36} - stage 1	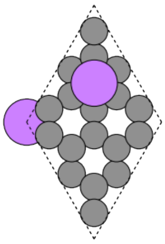	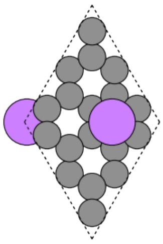	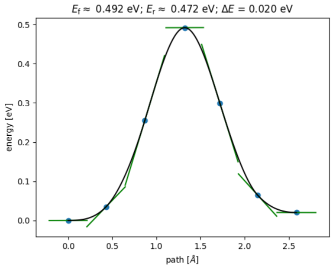

Table 3: NEBS - continuation 2

**Structure**	**Initial State**	**Final State**	**Barrier**
Li_4C_{36} - stage 1	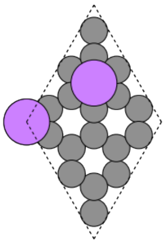	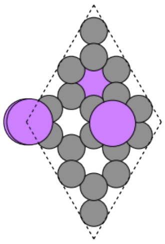	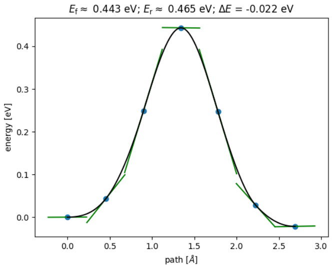
$Li_4C_{36}*$ - stage 1	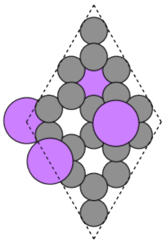	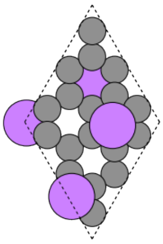	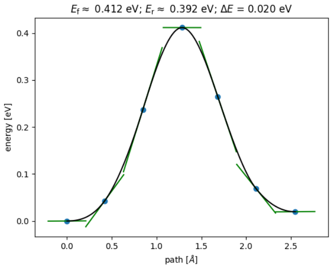
Li_6C_{36} - stage 1	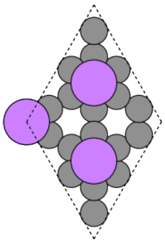	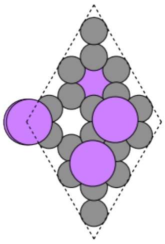	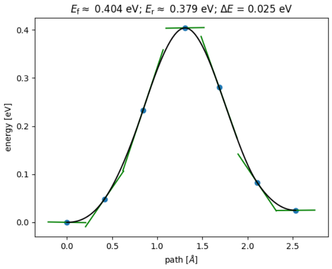
$Li_6C_{36}*$ - stage 1	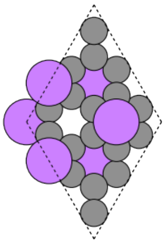	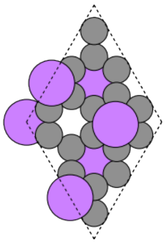	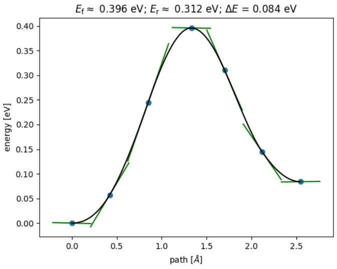

2 Machine-learned repulsive potentials

2.1 Exemplary training structures for GPrep

Several trajectories of Li clusters and bulk snippets were simulated with MD (timestep=5 fs, steps=2000). As starting structures served clusters with bcc structure (9 atoms), cuboctahedral (13 atoms), cubic (8 atoms), isoctahedral (13 atoms) and octahedral (19 atoms) shape as well as bulk Li (27 atoms) with periodic boundary conditions. The simulations were performed either at an average temperature of 300 K or 1000 K and every 100 timesteps was saved. In order to get also snapshots with smaller bond length, the starting structures were also compressed by factors between 0.75 and 1 in 10 steps and saved in another trajectory. Exemplary structures are shown in Figure 1. In addition to these, structures extracted from geometry relaxation pathways of LiC_2 and $LiC_{1.75}$ were also included, some of which randomly rattled. All the above were added to the training structures used for our previous parametrization.

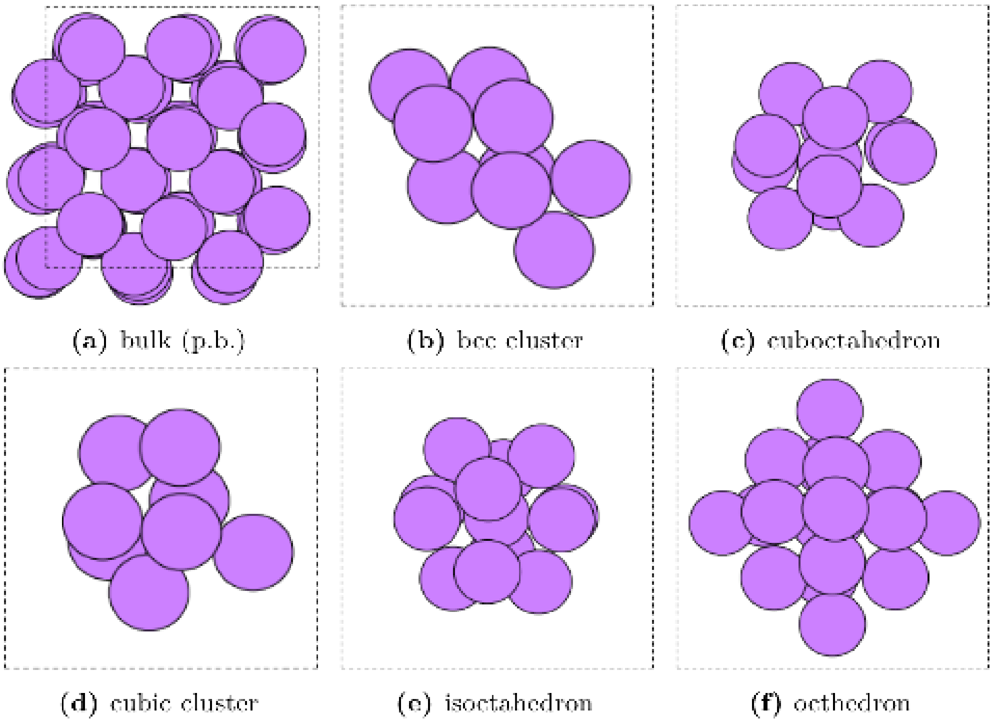

Figure 1: Last snapshot of MD trajectories (timestep=5 fs, steps=2000, T=300 K, NVE) of the used starting structures. For the bulk (bcc), periodic boundary conditions were applied. Some structures show more other less displacements, corresponding to more or less stable starting structures.

The full training set is available upon request.

2.2 Resulting potentials

Table 4 shows the GPR hyperparameters for the present parametrization compared to the previous one.

Table 4: GPR Hyperparameters for the present and previous parametrization

		C-C			Li-C			Li-Li		
	σ	β	θ	R_{cut}	β	θ	R_{cut}	β	θ	R_{cut}
GPrep previous	0.02	1.0	0.8	2.2	1.0	0.8	5.0	—	—	—
GPrep present	0.10	3.0	0.6	2.2	1.0	0.8	5.0	2.0	0.6	4.0

Figures 2 3 and 4 show the resulting GPrep potentials as well as a comparison with our previous parametrization. The C-C repulsion only changed very little and, correspondingly, the prediction of properties of empty graphite is not affected. Conversely, the Li-C potential exhibits more significant differences. Interestingly, only the inclusion of superdense $LiC_{6\text{-}x}$ training structures allows to capture the correct relative energetics over the entire range of states of charge. Properties that were already correctly predicted with the previous parametrization (*e.g.* layer spacings) were not affected.

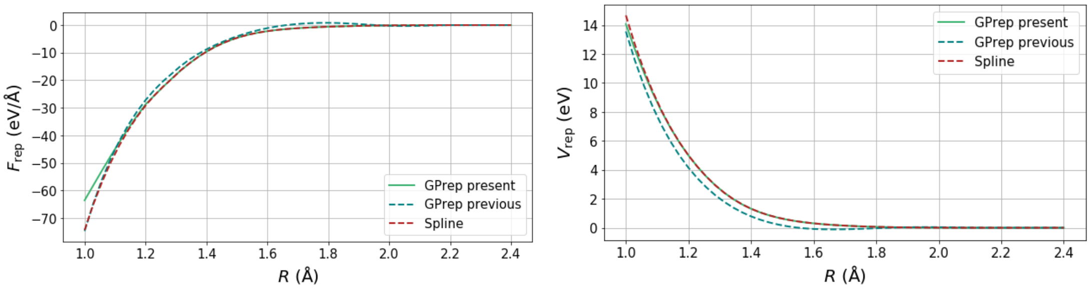

Figure 2: Repulsive force (left) and potential (right) obtained with GPrep for the C-C interaction. The shape did not change significantly with respect the previous parametrization.

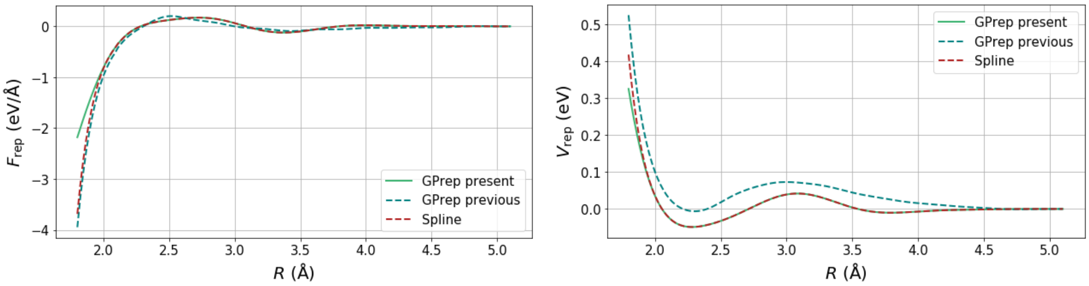

Figure 3: Repulsive force (left) and potential (right) obtained with GPrep for the Li-C interaction. The shape changed significantly with respect the previous parametrization.

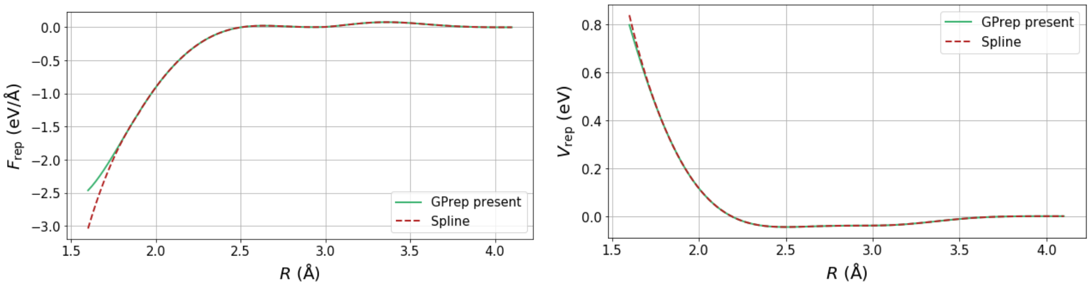

Figure 4: Repulsive force (left) and potential (right) obtained with GPrep for the Li-Li interaction. This potential was set to zero in the previous parametrization.

2.3 Li-Li forcematching

Figure 5 shows an exemplary validation force matching using structures extracted from NEB paths and from MD snapshots of bulk bcc lithium at 1000K. The forces are color-coded by their physical significance. The model shows a non-negligible scattering for metallic lithium, however this did not affect the calculated properties so far.

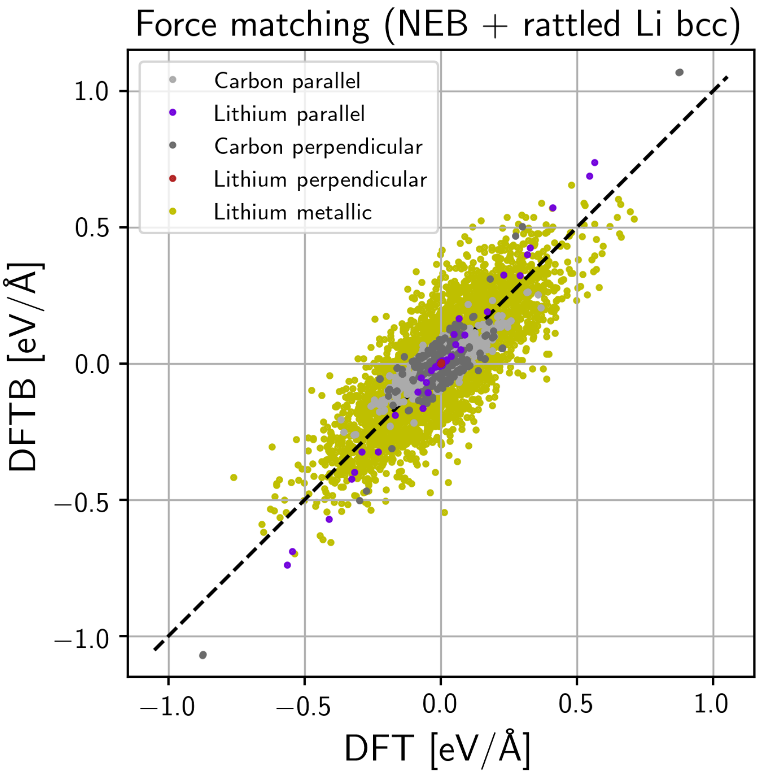

Figure 5: Force matching GPrep-DFTB vs DFT for selected validation structures (not included in the training set), extracted from NEB paths and from MD snapshots of bulk bcc lithium at 1000K.
