# Supplementary figures and images for: Accessing Structural, Electronic, Transport and Mesoscale Properties of Li-GICs via a Complete DFTB Model with Machine-Learned Repulsion Potential
Source: Materials (Basel). 2021 Nov 3;14(21):6633. doi: 10.3390/ma14216633 (PMC8585443; doi:10.3390/ma14216633)

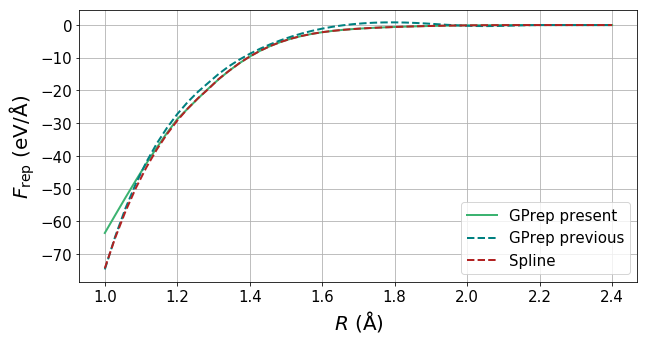

Supplement: Supplementary file 1 [file materials-14-06633-s001.zip › materials-1424747-supplementary/SI/images/CC_Frep.png]

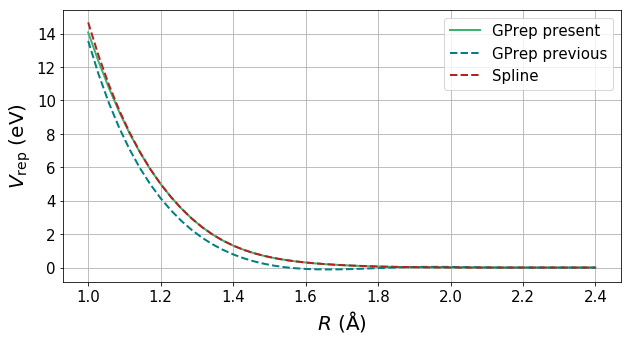

Supplement: Supplementary file 1 [file materials-14-06633-s001.zip › materials-1424747-supplementary/SI/images/CC_Vrep.png]

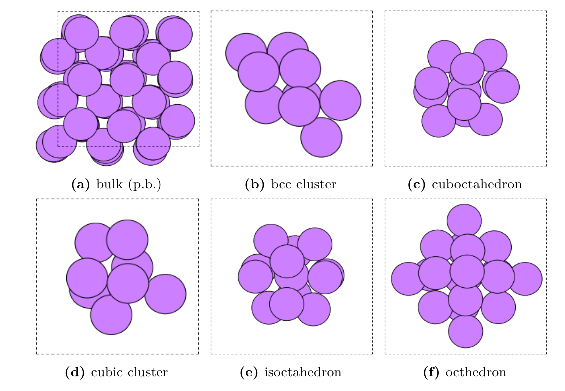

Supplement: Supplementary file 1 [file materials-14-06633-s001.zip › materials-1424747-supplementary/SI/images/clu.png]

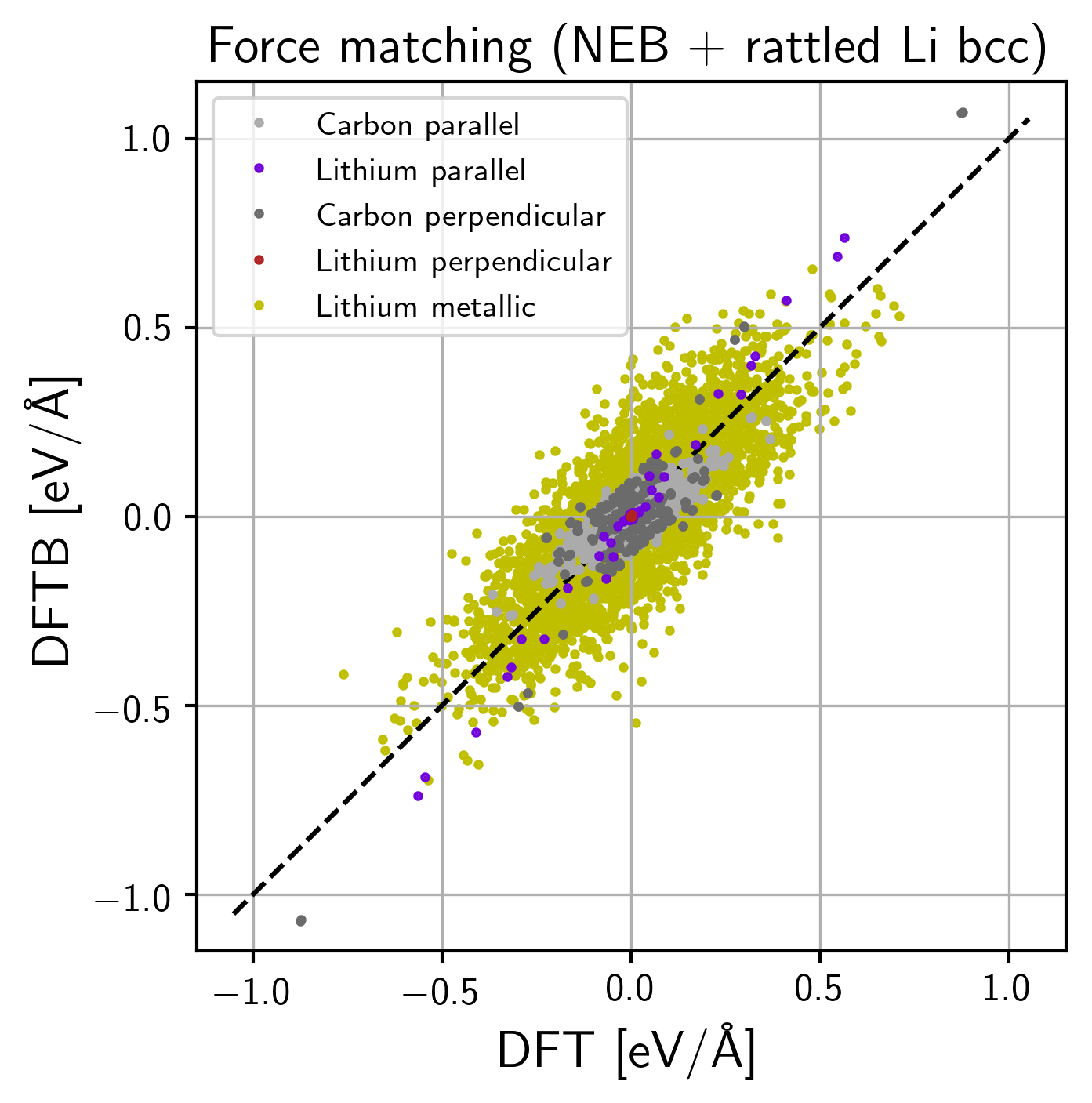

Supplement: Supplementary file 1 [file materials-14-06633-s001.zip › materials-1424747-supplementary/SI/images/forcematch.png]

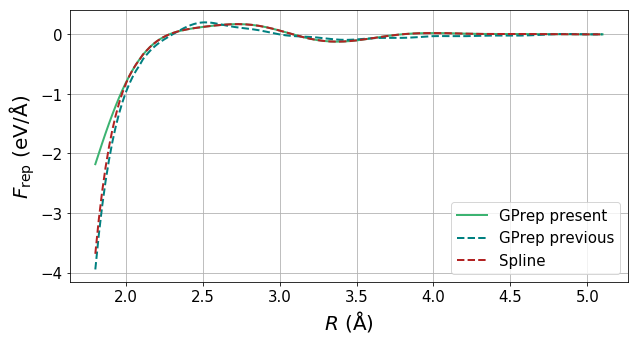

Supplement: Supplementary file 1 [file materials-14-06633-s001.zip › materials-1424747-supplementary/SI/images/LiC_Frep.png]

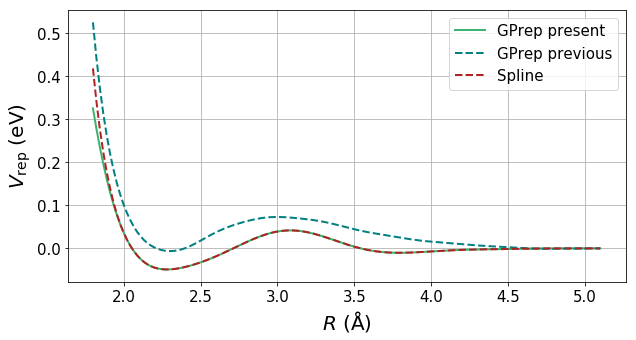

Supplement: Supplementary file 1 [file materials-14-06633-s001.zip › materials-1424747-supplementary/SI/images/LiC_Vrep.png]

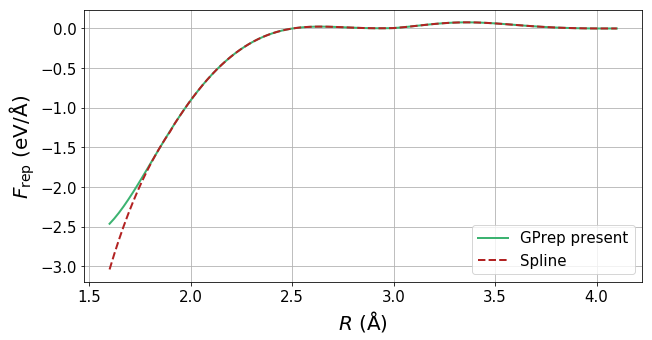

Supplement: Supplementary file 1 [file materials-14-06633-s001.zip › materials-1424747-supplementary/SI/images/LiLi_Frep.png]

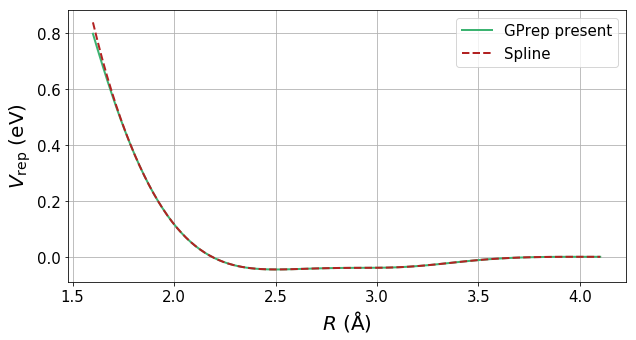

Supplement: Supplementary file 1 [file materials-14-06633-s001.zip › materials-1424747-supplementary/SI/images/LiLi_Vrep.png]

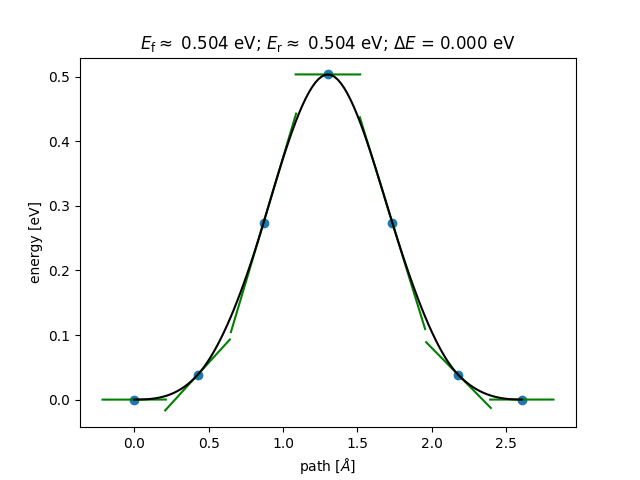

Supplement: Supplementary file 1 [file materials-14-06633-s001.zip › materials-1424747-supplementary/SI/images/sup3_Li1C36_stage2_diffusion-barrier.png]

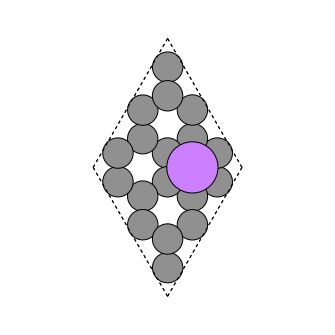

Supplement: Supplementary file 1 [file materials-14-06633-s001.zip › materials-1424747-supplementary/SI/images/sup3_Li1C36_stage2_FS.png]

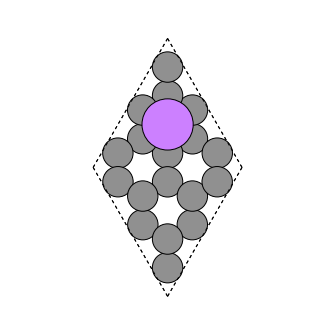

Supplement: Supplementary file 1 [file materials-14-06633-s001.zip › materials-1424747-supplementary/SI/images/sup3_Li1C36_stage2_IS.png]

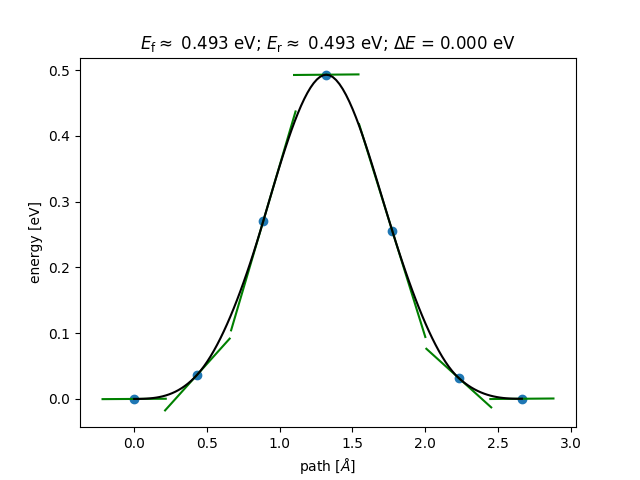

Supplement: Supplementary file 1 [file materials-14-06633-s001.zip › materials-1424747-supplementary/SI/images/sup3_Li1C48_stage3_diffusion-barrier.png]

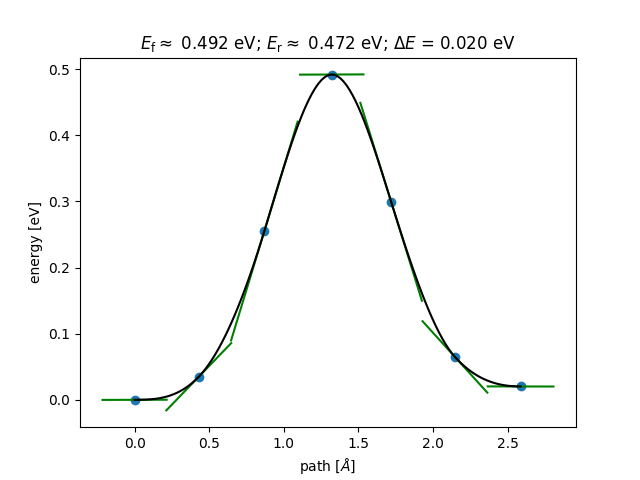

Supplement: Supplementary file 1 [file materials-14-06633-s001.zip › materials-1424747-supplementary/SI/images/sup3_Li2C36_stage1_diffusion-barrier.png]

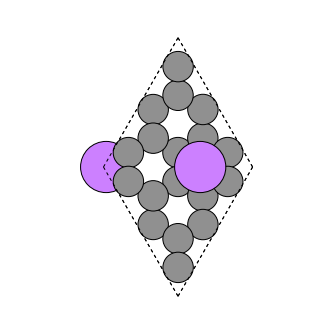

Supplement: Supplementary file 1 [file materials-14-06633-s001.zip › materials-1424747-supplementary/SI/images/sup3_Li2C36_stage1_FS.png]

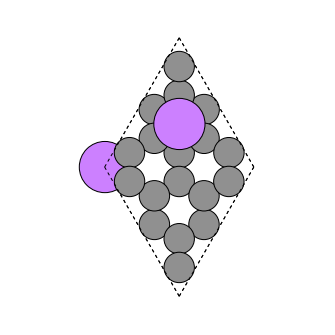

Supplement: Supplementary file 1 [file materials-14-06633-s001.zip › materials-1424747-supplementary/SI/images/sup3_Li2C36_stage1_IS.png]

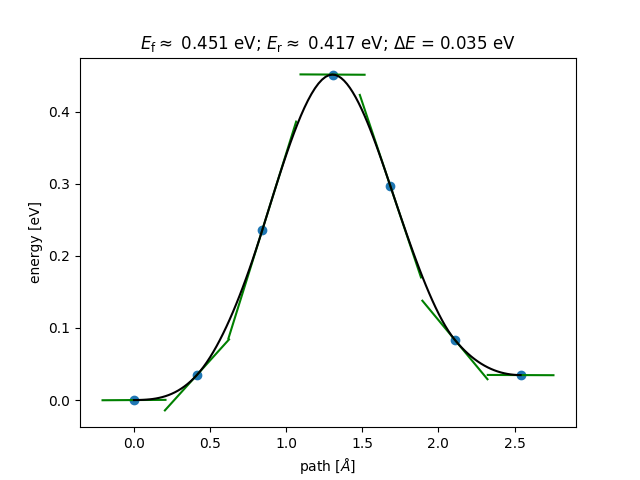

Supplement: Supplementary file 1 [file materials-14-06633-s001.zip › materials-1424747-supplementary/SI/images/sup3_Li2C36_stage2_diffusion-barrier.png]

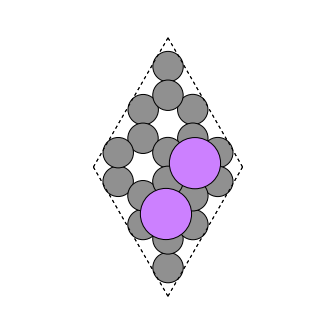

Supplement: Supplementary file 1 [file materials-14-06633-s001.zip › materials-1424747-supplementary/SI/images/sup3_Li2C36_stage2_FS.png]

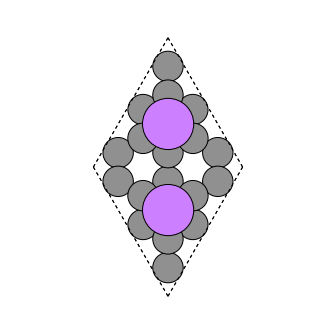

Supplement: Supplementary file 1 [file materials-14-06633-s001.zip › materials-1424747-supplementary/SI/images/sup3_Li2C36_stage2_IS.png]

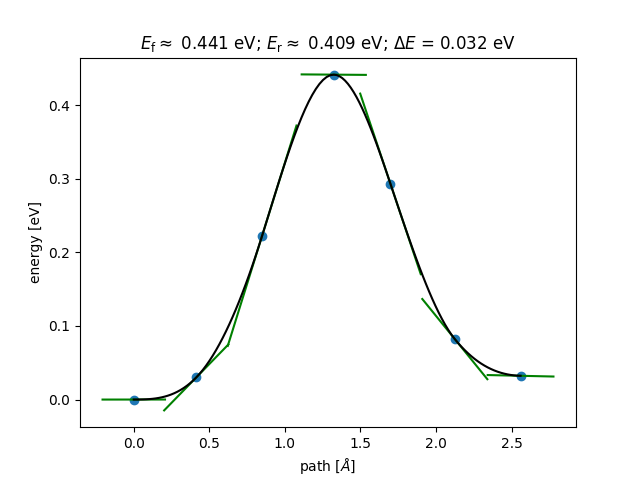

Supplement: Supplementary file 1 [file materials-14-06633-s001.zip › materials-1424747-supplementary/SI/images/sup3_Li2C48_stage3_diffusion-barrier.png]

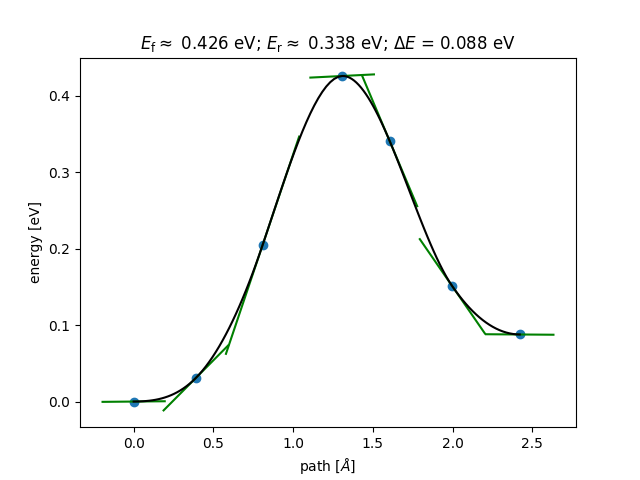

Supplement: Supplementary file 1 [file materials-14-06633-s001.zip › materials-1424747-supplementary/SI/images/sup3_Li3C36_stage2_diffusion-barrier.png]

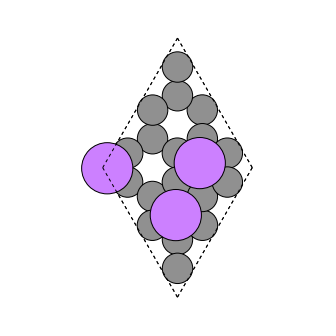

Supplement: Supplementary file 1 [file materials-14-06633-s001.zip › materials-1424747-supplementary/SI/images/sup3_Li3C36_stage2_FS.png]

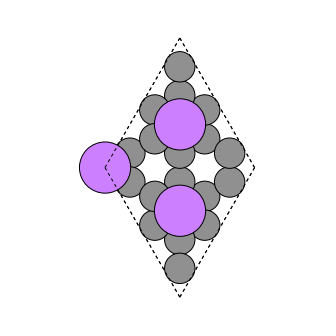

Supplement: Supplementary file 1 [file materials-14-06633-s001.zip › materials-1424747-supplementary/SI/images/sup3_Li3C36_stage2_IS.png]

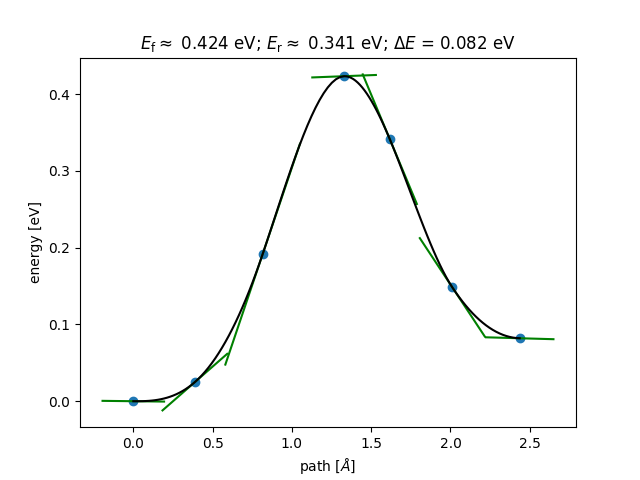

Supplement: Supplementary file 1 [file materials-14-06633-s001.zip › materials-1424747-supplementary/SI/images/sup3_Li3C48_stage3_diffusion-barrier.png]

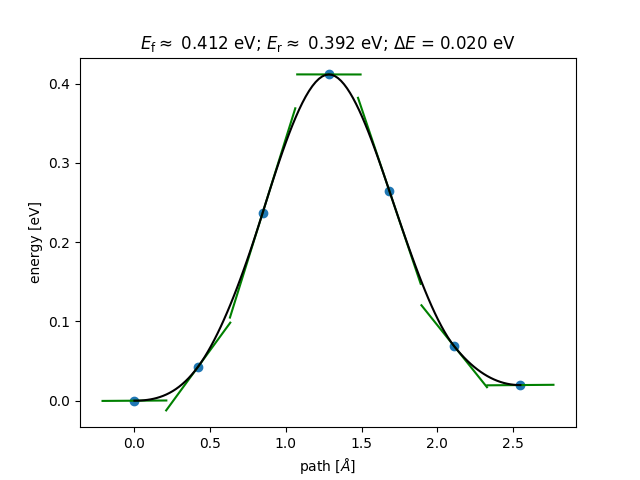

Supplement: Supplementary file 1 [file materials-14-06633-s001.zip › materials-1424747-supplementary/SI/images/sup3_Li4C36_AB_diffusion-barrier.png]

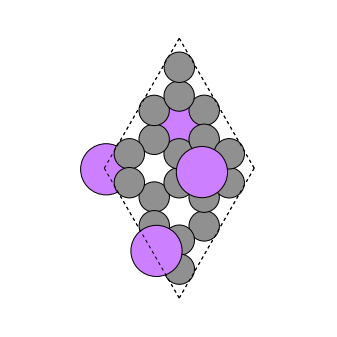

Supplement: Supplementary file 1 [file materials-14-06633-s001.zip › materials-1424747-supplementary/SI/images/sup3_Li4C36_AB_FS.png]

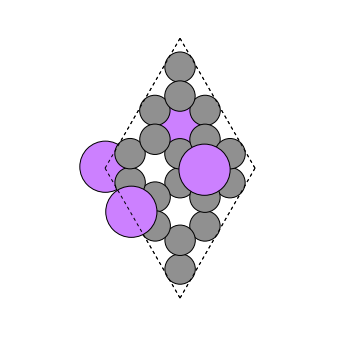

Supplement: Supplementary file 1 [file materials-14-06633-s001.zip › materials-1424747-supplementary/SI/images/sup3_Li4C36_AB_IS.png]

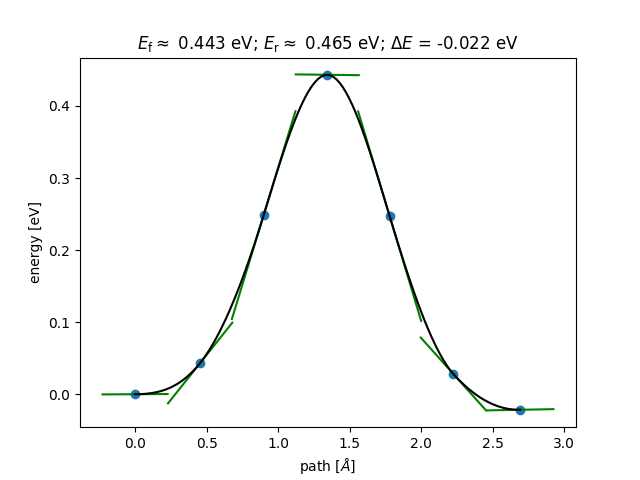

Supplement: Supplementary file 1 [file materials-14-06633-s001.zip › materials-1424747-supplementary/SI/images/sup3_Li4C36_diffusion-barrier.png]

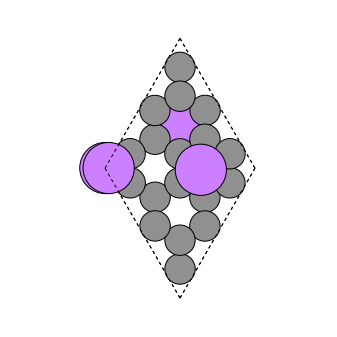

Supplement: Supplementary file 1 [file materials-14-06633-s001.zip › materials-1424747-supplementary/SI/images/sup3_Li4C36_FS.png]

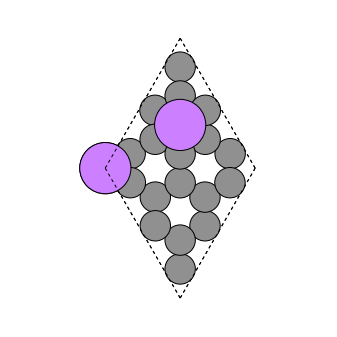

Supplement: Supplementary file 1 [file materials-14-06633-s001.zip › materials-1424747-supplementary/SI/images/sup3_Li4C36_IS.png]

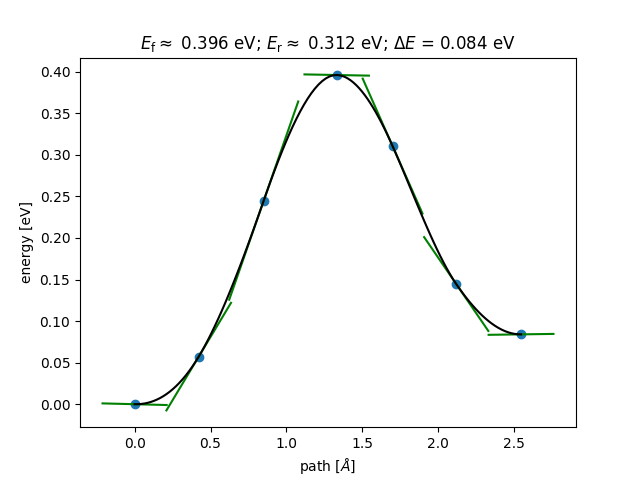

Supplement: Supplementary file 1 [file materials-14-06633-s001.zip › materials-1424747-supplementary/SI/images/sup3_Li6C36_AB_diffusion-barrier.png]

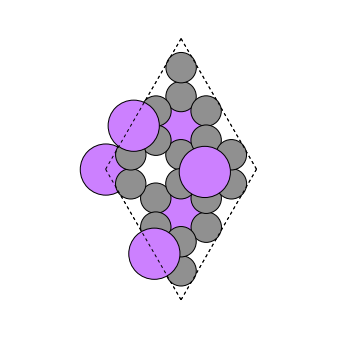

Supplement: Supplementary file 1 [file materials-14-06633-s001.zip › materials-1424747-supplementary/SI/images/sup3_Li6C36_AB_FS.png]

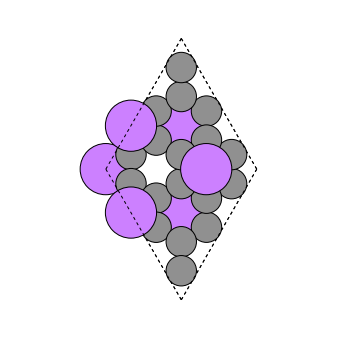

Supplement: Supplementary file 1 [file materials-14-06633-s001.zip › materials-1424747-supplementary/SI/images/sup3_Li6C36_AB_IS.png]

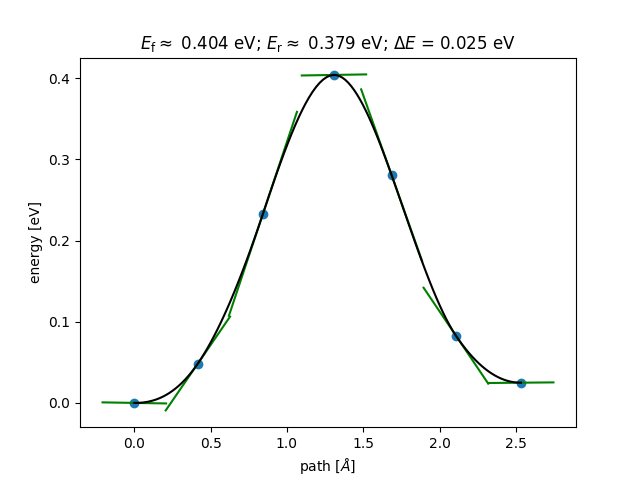

Supplement: Supplementary file 1 [file materials-14-06633-s001.zip › materials-1424747-supplementary/SI/images/sup3_Li6C36_diffusion-barrier.png]

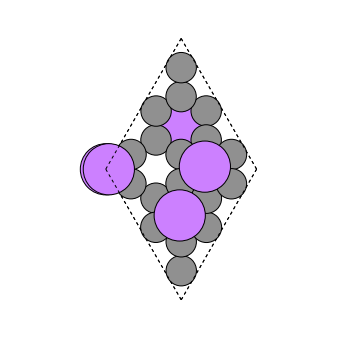

Supplement: Supplementary file 1 [file materials-14-06633-s001.zip › materials-1424747-supplementary/SI/images/sup3_Li6C36_FS.png]

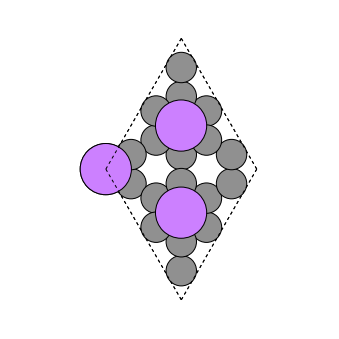

Supplement: Supplementary file 1 [file materials-14-06633-s001.zip › materials-1424747-supplementary/SI/images/sup3_Li6C36_IS.png]

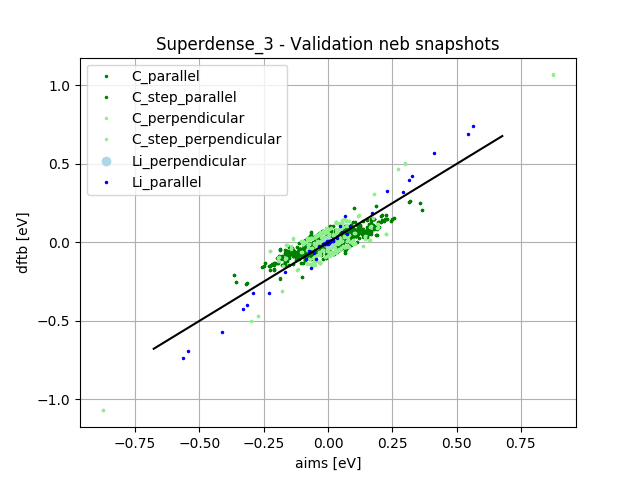

Supplement: Supplementary file 1 [file materials-14-06633-s001.zip › materials-1424747-supplementary/SI/images/validation.png]
